# Role of Clathrin Assembly Protein-2 Beta Subunit during White Spot Syndrome Virus Infection in Black Tiger Shrimp *Penaeus monodon*

**DOI:** 10.1038/s41598-019-49852-0

**Published:** 2019-09-17

**Authors:** Thapanan Jatuyosporn, Pasunee Laohawutthichai, Premruethai Supungul, Rogerio R. Sotelo-Mundo, Adrian Ochoa-Leyva, Anchalee Tassanakajon, Kuakarun Krusong

**Affiliations:** 1 Structural and Computational Biology Research Unit, Department of Biochemistry, Faculty of Science, Chulalongkorn University, Bangkok, 10330 Thailand; 2 Center of Excellence for Molecular Biology and Genomics of Shrimp, Department of Biochemistry, Faculty of Science, Chulalongkorn University, Bangkok, 10330 Thailand; 3 National Center for Genetic Engineering and Biotechnology (BIOTEC), National Science and Technology Development Agency (NSTDA), Pathumthani, 12120 Thailand; 4 Laboratorio de Estructura Biomolecular, Centro de Investigación en Alimentación y Desarrollo, A.C. (CIAD), Carretera Gustavo Enrique Astiazaran Rosas No. 46, Hermosillo, Sonora 83304 Mexico; 5 Departamentos de Microbiología Molecular, Medicina Molecular y Bioprocesos, Unidad Universitaria de Secuenciacián Masiva y Bioinformática, Instituto de Biotecnología (IBT), Universidad Nacional Autónoma de México (UNAM), Avenida Universidad 2001, Colonia Chamilpa, Cuernavaca 62210 Mexico

**Keywords:** Immunology, Ocean sciences, Pathogens

## Abstract

White spot syndrome virus (WSSV) is one of the most lethal viruses severely affecting shrimp industry. This disease can cause 100% mortality of farmed shrimp within a week. This work aims to characterize clathrin assembly proteins in *Penaeus monodon* and investigate their roles in WSSV entry. In general, clathrin assembly proteins form complexes with specific receptors and clathrins, leading to clathrin-mediated endocytosis. Adaptor protein 2 (AP-2), which is responsible for endocytosis at plasma membrane, consists of four subunits including α, β2, μ2 and σ2. Knockdown of clathrin coat AP17, or σ subunit of AP-2 dramatically reduced WSSV infectivity. Similar results were observed, when shrimp were pre-treated with chlorpromazine (CPZ), an inhibitor of clathrin-dependent endocytosis. The complete open reading frames of AP-2β and μ subunits of *P*. *monodon* are reported. *Pm*AP-2 β was up-regulated about 4-fold at 6 and 36 h post-WSSV infection. Knockdown of *Pm*AP-2β delayed shrimp mortality during WSSV infection, of which WSSV intermediate early 1 gene expression was also down-regulated. Immunogold-labelling and transmission electron microscopy revealed that *Pm*AP-2β co-localized with WSSV particles at plasma membrane. In addition, *Pm*AP-2β-silencing significantly affected the expression levels of *Pm*STAT, *Pm*DOME, *Pm*Dorsal and ALF*Pm*3 during WSSV infection. It is possible that *Pm*AP-2β is associated with the JAK/STAT and the Toll pathway.

## Introduction

White spot syndrome virus (WSSV) is a highly pathogenic virus that causes massive death in penaeid shrimp. Once infected by WSSV, 100% cumulative mortality of farmed shrimps could be expected within 3–10 days^1^. WSSV is a non-occluded, enveloped, rod-shaped, double stranded DNA virus, which belongs to the genus *Whispovirus* and family *Nimaviridae*^2,3^. The rod-shaped, intact virus particle is about 70–167 nm x 210–380 nm^4–8^ and contains the genome of approximately 300 kbp, depending on the viral isolate^9–11^. Several reports have suggested clathrin-mediated endocytosis involves in WSSV infection in the pacific white shrimp *Litopenaeus vannamei*^12^ and the red claw crayfish *Cherax quadricarinatus* hematopoietic tissue cells^13,14^. Both DNA viruses, such as African swine fever virus, Vaccinia virus and Singapore Grouper Iridovirus, and RNA viruses, including Ebolavirus, Hepatitis C virus, Influenza A virus, Dengue virus and Yellow head virus, are internalized via clathrin-dependent endocytosis^15–22^.

Clathrin-mediated endocytosis is a well-characterized process responsible for the transportation of a wide variety of molecules from the cell surface inside the cells. Clathrin-adaptor protein 2 (AP-2) is responsible for endocytosis at the plasma membrane while AP-1 and AP-3 complexes participate in endocytic vesicle formation at the trans-Golgi network and at the membrane of lysosomes, respectively^23^. In general, AP-2 complex consists of 4 subunits: 2 large subunits (α and β), one medium subunit (µ) and one small subunit (σ). The α subunit recruits AP-2 complex to plasma membrane by interacting with phosphatidylinositol 4,5-bisphosphate (PI(4,5)P_2_)^24^, while the β2 subunit links AP-2 with clathrin and may play a role in the selection of specific cargo^25^. The μ2 subunit recognizes and sorts protein cargo^26^. The well-characterized sorting signals within transmembrane cargo molecules are tyrosine-based (YXXØ) and dileucine-based [DE]XXXL[LI] signals, when X is any amino acid residues, Ø represents a bulky hydrophobic amino acid and the brackets indicates either amino acid is allowed at that position^27^. Tyrosine-based signals interact with AP2 complexes through the binding with μ2, while dileucine-based signals bind to α-σ2 subunits. Previously, the σ subunit of AP-2, known as AP17 in *P*. *monodon*, has been characterized^22^.

This study reports, for the first time, two main components of AP-2 complexes in *P*. *monodon*, *Pm*AP-2β and *Pm*AP2-μ, which play a part in cargo recognition. RNA interference technique, endocytosis inhibitor, immunofluorescence confocal microscopy and transmission electron microscopy (TEM) were used to investigate WSSV infection via clathrin-dependent endocytosis.

## Results

### Silencing of *Pm*-clathrin coat AP17 and chlorpromazine (CPZ) treatment reduced WSSV propagation

**Figure 1:**
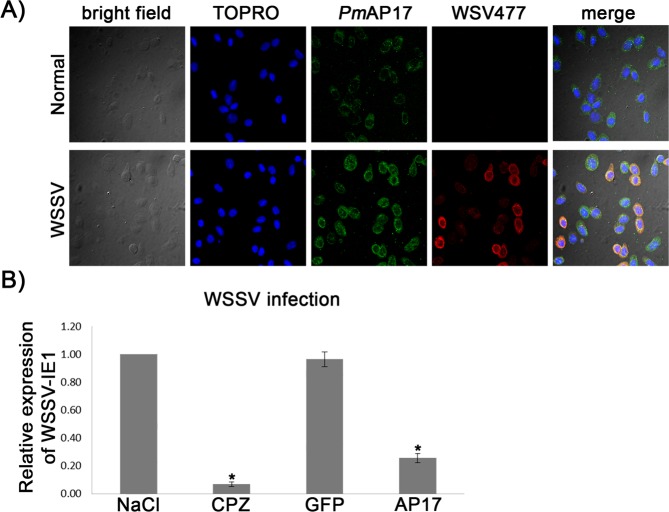
Effect of *Pm*-clathrin coat AP17 silencing and chlorpromazine on WSSV propagation. (**A**) Immunofluorescent staining analysis of *Pm*-clathrin coat AP17 and WSV477 in hemocytes by confocal laser scanning microscopy. Hemocytes from unchallenged and WSSV challenged were collected at 24 hpi and probed by anti-*Pm*-clathrin coat AP17 and WSV477 antibodies. *Pm*-clathrin coat AP17 and WSV477 proteins were visualized by secondary antibodies conjugated with Alexa Fluor 488 (green) and Alexa Fluor 568 (red), respectively. Nuclei were stained in blue. (**B**) The evaluated replicativity of WSSV in *Pm*-clathrin coat AP17 silencing and chlorpromazine pre-treated shrimp. Shrimp were divided into four groups and injected with either 150 mM NaCl, GFP dsRNA (10 µg/g shrimp), *Pm*-clathrin coat AP17 dsRNA (10 µg/g shrimp) or chlorpromazine (0.25 µg/g shrimp). After WSSV infection, the hemocyte were collected and used for determination of WSSV replicativity. The mRNA expression levels of WSSV IE1 were analyzed by Quantitative Real-time RT-PCR. The data are shown as the mean ± standard deviation. An asterisk represents significant differences from control group (*p* < 0.05). The experiment was carried out in triplicates.

Immunofluorescence confocal microscopy showed that *Pm*-clathrin coat AP17 was found in the cytoplasm of shrimp hemocytes and the level of *Pm*-clathrin coat AP17 was higher in WSSV infected shrimp hemocytes, in comparison to non-infected shrimp (Fig. 1A). *Pm*-clathrin coat AP17 (green) was also localized with WSV477 (red) in the cytoplasm of shrimp hemocytes. Silencing of *Pm*-clathrin coat AP17 and CPZ pre-treatment resulted in a significant reduction of WSSV-IE1 transcripts at 12 hpi (Fig. 1B). *Pm*-clathrin coat AP17 gene knockdown lower WSSV-IE1 expression by 4.3-fold, while CPZ treatment reduced WSSV-IE1 transcription level by 12.5-fold. Both suggested that clathrin-mediated endocytosis plays a role in WSSV entry.

### Gene cloning, bioinformatics analysis and recombinant protein production of *Pm*AP-2β and *Pm*AP-2μ

**Figure 2:**
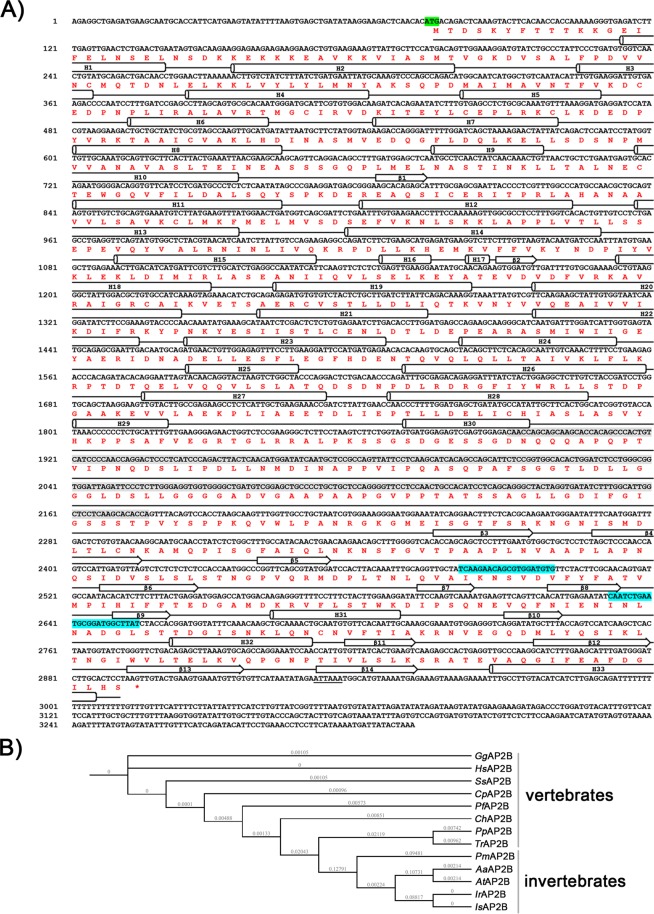
Analysis of nucleotide and amino acid sequences of *Pm*AP-2β. (**A**) Nucleotide and amino acid sequence of *Pm*AP-2β. Start codon is in a box and polyadenylation signal is underlined. Secondary structure prediction was carried out using Jpred4^61^. (**B**) Phylogenetic tree of AP-2β from invertebrates and vertebrates. Sequences of AP-2β were obtained from The National Center for Biotechnology Information (NCBI) and phylogenetic tree was generated by iTOL. AP-2βs are from *Gorilla gorilla gorilla* (*Gg*AP2B, G3RHU1); *Homo sapiens* (*Hs*AP2B, P63010); *Sus scrofa* (*Ss*AP2B,acc. NO.NP_001230112.1); *Cavia porcellus* (*Cp*AP2B, acc. NO.XP_003469646.1); *Patagioenas fasciata monilis* (*Pf*AP2B, acc.NO. OPJ78715.1); *Crotalus horridus* (*Ch*AP2B, acc. NO.GAAZ01000182.1); *Poeciliopsis prolifica* (*Pp*AP2B, acc. NO.GBYX01461436.1); *Takifugu rubripes* (*Tr*AP2B, acc. NO.XP_003970865.1); *Penaeus monodon* (*Pm*AP2B); *Anopheles albimanus* (*Aa*AP2B, acc. NO.A0A182FRP2); *Anopheles triannulatus* (*At*AP2B, acc. NO.GGFK01000869.1); *Ir*, *Ixodes Ricinus* (acc. NO.GEFM01002526.1); *Is*, *Ixodes scapularis* (acc. NO. XP_002412040.1).

To further investigate role of clathrin-mediated endocytosis during WSSV infection, other clathrin adaptor proteins, *Pm*AP-2β and *Pm*AP-2μ were studied. The complete 2,820 bp *Pm*AP-2β cDNA sequence was obtained from the *P*. *monodon* EST database (http://pmonodon.biotec.or.th). The open reading frame (ORF) of *Pm*AP-2β (GenBank accession number MK089559) can be translated into a protein of 939 amino acid residues (Fig. 2A), with a predicted molecular weight of 103 kDa and pI of 4.98.

**Figure 3:**
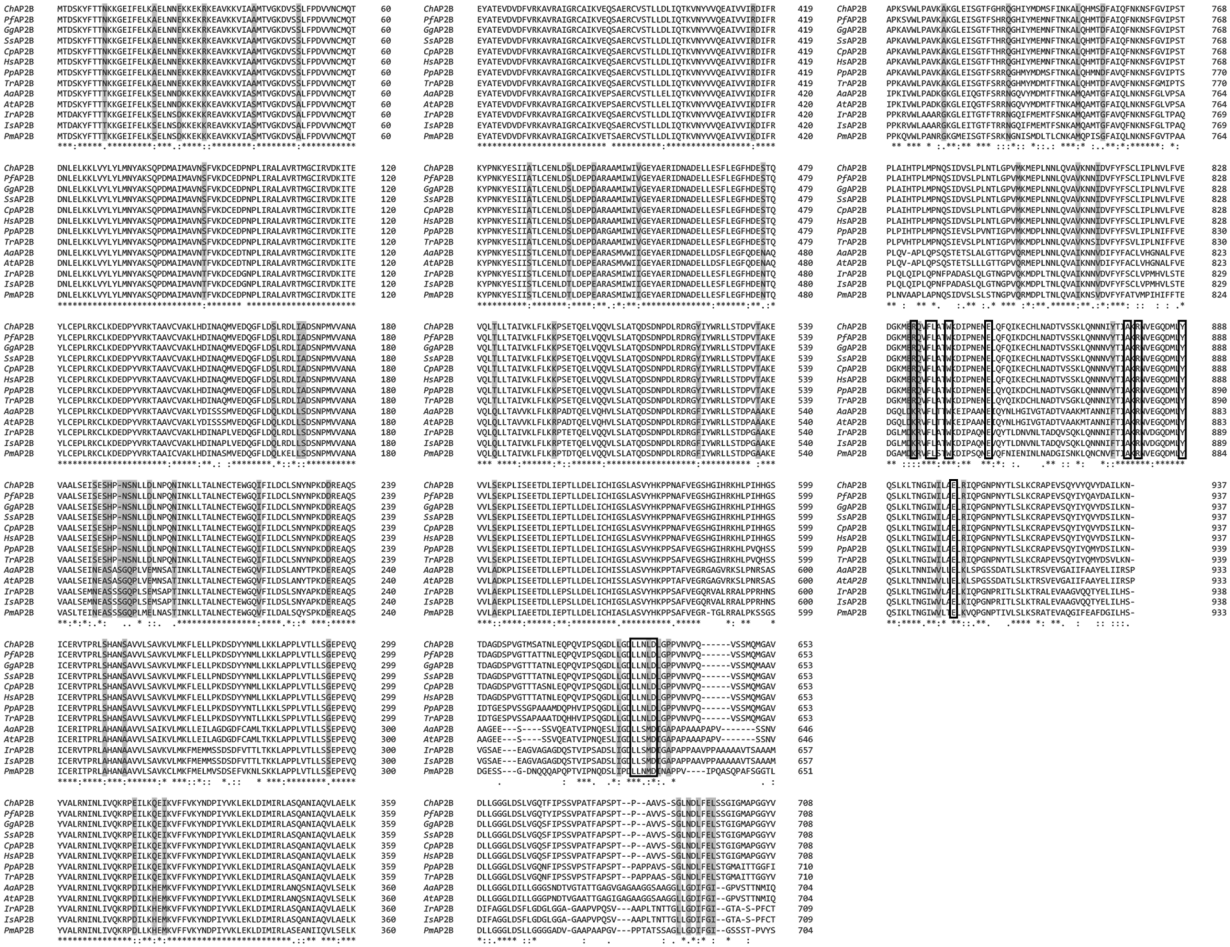
Multiple sequence alignment of AP-2β from invertebrates and vertebrates. AP-2βs are from *Crotalus horridus* (*Ch*AP2B, acc. NO.GAAZ01000182.1); *Patagioenas fasciata monilis* (*Pf*AP2B, acc.NO. OPJ78715.1); *Gorilla gorilla gorilla* (*Gg*AP2B, G3RHU1); *Sus scrofa* (*Ss*AP2B, acc. NO.NP_001230112.1); *Cavia porcellus* (*Cp*AP2B, acc. NO.XP_003469646.1); *Homo sapiens* (*Hs*AP2B, P63010); *Poeciliopsis prolifica* (*Pp*AP2B, acc. NO.GBYX01461436.1); *Takifugu rubripes* (*Tr*AP2B, acc. NO.XP_003970865.1); *Anopheles albimanus* (*Aa*AP2B, acc. NO.A0A182FRP2); *Anopheles triannulatus* (*At*AP2B, acc. NO.GGFK01000869.1); *Ir*, *Ixodes Ricinus* (acc. NO.GEFM01002526.1); *Is*, *Ixodes scapularis* (acc. NO. XP_002412040.1); *Penaeus monodon* (*Pm*AP2B).

The deduced amino acid sequence of *Pm*AP-2β showed high sequence similarity to the putative AP-2 complex subunit β-1 from *Litopenaeus vannamei* (ROT82409.1, 98% identity), followed by *Pm*AP-2β from *Ixodes* species (acc. NO.GEFM01002526.1 and XP_002412040.1, 81.1% identity) and from *Anopheles* species (acc. NO. A0A182FRP2 and GGFK01000869.1, 78.2% identity). The phylogenetic trees of AP-2 complex subunit β from vertebrates and invertebrates are displayed in Fig. 2B. Multiple sequence alignment suggests amino acid differences in 60 positions that distinguish AP-2 complex subunit β in vertebrates from those in invertebrates (Fig. 3).

Previously, the crystal structure of *Homo sapiens* AP-2β revealed that the protein possesses three domains, including the N-terminal trunk domain, the C-terminal appendage domain and the flexible hinge region located between N- and C-terminal domains^28^. The N-terminal domain of *Pm*AP-2β shares strong sequence identity (82.53%) with *Hm*AP-2β^28^, however, the C-terminal domain has less sequence identity but shows similar structural fold^29^. In *Homo sapiens*, C-terminal appendages bind with [DE]_n_X_1–2_FXX[FL]XXXR motif in clathrin-coated accessory proteins such as Epsin 1, Epsin 2, β-arrestin 1, β-arrestin 2 and autosomal recessive hypercholesterolemia (ARH) via R834, F837, L838, W841, E849, A877, R879, Y888 and E902. These amino acid residues are highly conserved in invertebrates, but Arginine at position 834 (according to *Hm*AP-2β) is replaced by Lysine residue in invertebrates. The flexible hinge region has been reported to interact with N-terminal of clathrin heavy chain using a canonical clathrin box motif (LLNLD)^30,31^, which is strongly conserved in vertebrates (Fig. 3). However, this motif in invertebrates is less conserved. It presents as LLSMD in insecta (*Ixodes Ricinus*, *Ixodes scapularis*, *Anopheles albimanus* and *Anopheles triannulatus*) and LLNMD in *P*. *monodon*.

*Pm*AP-2β was expressed in *Escherichia coli* BL21 CodonPlus and the recombinant protein was purified by Ni Sepharose 6 Fast Flow under denaturing condition and then refolded. In Fig. S1 in Supplementary Information, *Pm*AP-2β showed an apparent molecular weight of approximately 110 kDa, which is closed to the calculated molecular weight of His-tagged fusion *Pm*AP-2β.

In addition, 5′- Rapid Amplification of cDNA Ends (5′-RACE) was performed to obtain the full-length *Pm*AP-2μ cDNA sequence of 1,299-bp (GenBank accession number MK496174), which encodes 432 amino acids. *Pm*AP-2μ has calculated molecular weight of 49.3 kDa and pI of 9.55. In Fig. S1 in Supplementary Information, the recombinant His-tagged fusion *Pm*AP2μ was successfully purified as a single band with an apparent molecular weight of 53 kDa. Based on protein sequence analysis, *Pm*AP-2μ contains two major domains, including longin-like domain at the N-terminus and mu homology domain at the C-terminus. As shown in Fig. S2 in Supplementary Information, the conserved longin-like domain consists of β1, β2, H1, β2, β4, β5, H2 and H3, which interacts with AP-2β subunit to form clathrin assembly protein complexes^28^, while the mu homology domain has all β -sheet structure (β6-β23).

According to protein BLAST analysis and sequence homology search (BlastX), *Pm*AP2μ revealed high sequence similarity to the AP-2 complex subunit μ from *Litopenaeus vannamei* (XP_027231007.1, 99% identity), clathrin-associated adaptor protein complexes μ subunit from *C*. *quadricarinatus* (ALP46597.1, 98% identity), as well as AP-2 complex subunit μ from *Agrilus planipennis*, *Onthophagus taurus*, *Tribolium castaneum*, *Diabrotica virgifera virgifera* and *Anoplophora glabripennis* (XP_018335920.1, XP_022918165.1, NP_001280510.1, ATD50466.1 and XP_018578400.1, respectively, 91% identity). The phylogenetic trees revealed that AP-2 complex subunit μ can be divided into two main groups: vertebrates and invertebrates, and each of them contains two subgroups (Fig. S2). In invertebrates, AP-2μ is classified into Arthropoda subgroup (*P*. *monodon*, *L*. *vannamei* and *C*. *quadricarinatus*) and insect subgroup (*Nicrophorus vespilloides*, *Zootermopsis nevadensis* and *Bicyclus anynana*). In vertebrates, AP-2μ sequences from *Homo sapiens*, *Bos taurus* and *Mus musculus* are identical. Based on multiple sequence alignments, vertebrate and invertebrate AP-2μ can be distinguished by the amino acid residues at 14 different locations: two positions are on longin-like domain, while the other twelve locate on mu homology domain (Fig. S3 in Supplementary Information). T156 of *Homo sapiens* AP-2μ has been reported to be phosphorylated and essential for interacting with transferrin receptors *in vitro* and *in vivo *^32,33^, as well as for binding affinity of AP-2 complex to YXXɸ motif of the cargo proteins ^34,35^. This threonine corresponds to T154 in *P*. *monodon* and is highly conserved in all organisms. Moreover, seven crucial amino acid residues for vesicle traffic that interact to phosphoinositide lipids^ 25,28^, are also highly conserved across diverse phyta. These amino acid residues of *Homo sapiens* AP-2μ are K319, E321, K341, K343, K345, K354 and K356, corresponding to K316, E318, K338, K340, K342, K351 and K353 in *Pm*AP-2μ as shown in Fig. S3.

### *Analysis of Pm*AP-2β gene expression

**Figure 4:**
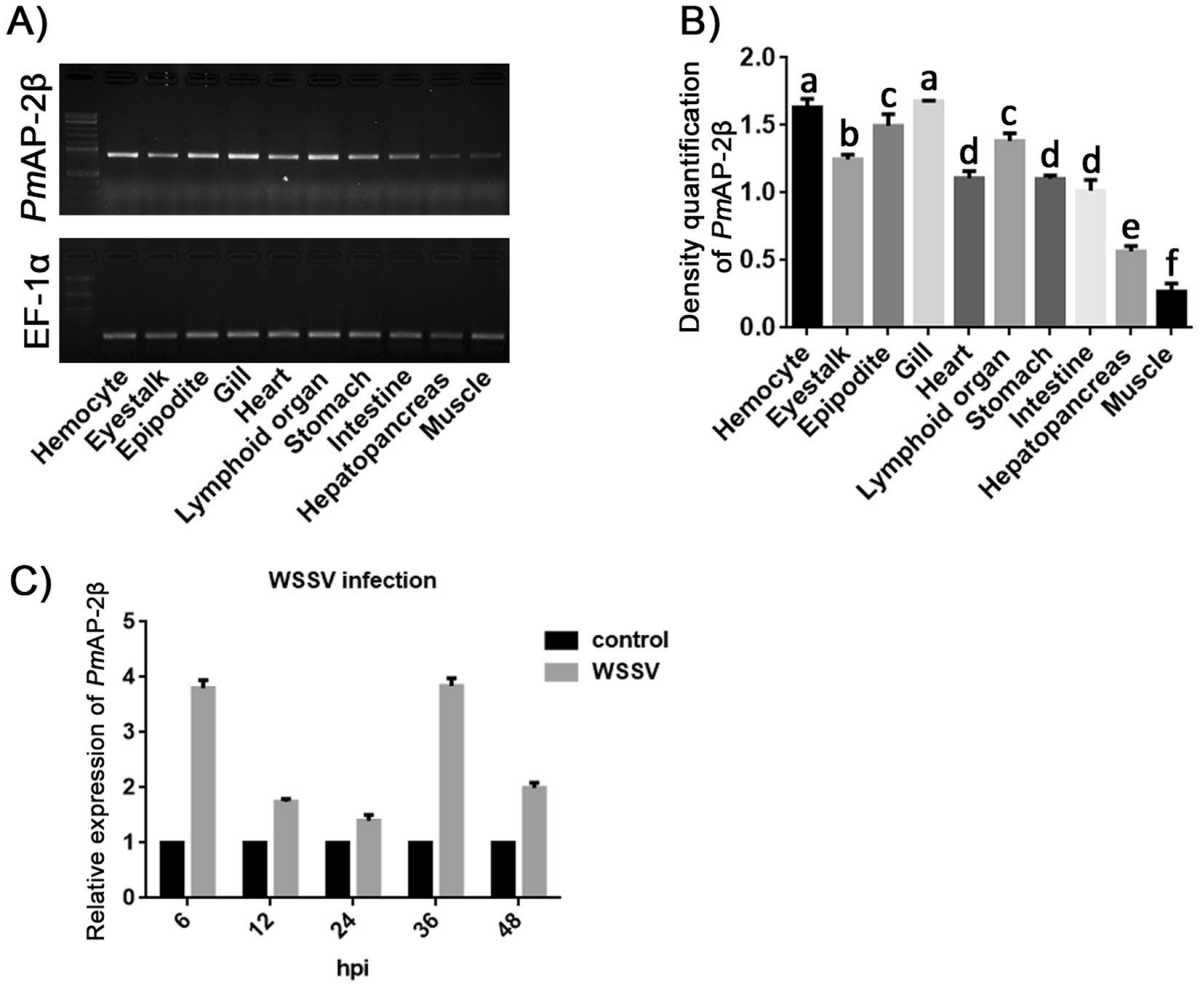
Analysis of *Pm*AP-2β expression in shrimp. (**A**) Tissue distribution of *Pm*AP-2β in hemocyte, eyestalk, epipodite, gill, heart, lymphoid organ, stomach, intestine, hepatopancreas and muscle. EF-1α was used as an internal control. (**B**) RT-PCR was used to determine *Pm*AP-2β gene expression in various shrimp tissues. The different letters above the bars represent significant differences at *p* < 0.05. (**C**) Relative expression ratio of *Pm*AP-2β gene in WSSV infected shrimp, analyzed by quantitative Real-time RT-PCR.

Ten tissues (hemocyte, eyestalk, epipodite, gill, heart, lymphoid organ, stomach, intestine, hepatopancreas and muscle) were collected to determine *Pm*AP-2β expression. The PCR products were analyzed by 1.5% agarose electrophoresis and measured intensity of *Pm*AP-2β and EF-1α (an internal control) by Gel Analyzer software. Among the tissues, *Pm*AP-2β was expressed at the highest level in hemocyte and gill, while the lowest amount of *Pm*AP-2β transcript was found in muscle (Fig. 4A,B). Fig. 4C showed that the expression level of *Pm*AP-2β in WSSV-infected shrimp was higher than that in unchallenged shrimp, especially at 6 and 36 hpi, at which *Pm*AP-2β was highly up-regulated for 4-fold.

### Silencing of *Pm*AP-2β lowered WSSV infection

**Figure 5:**
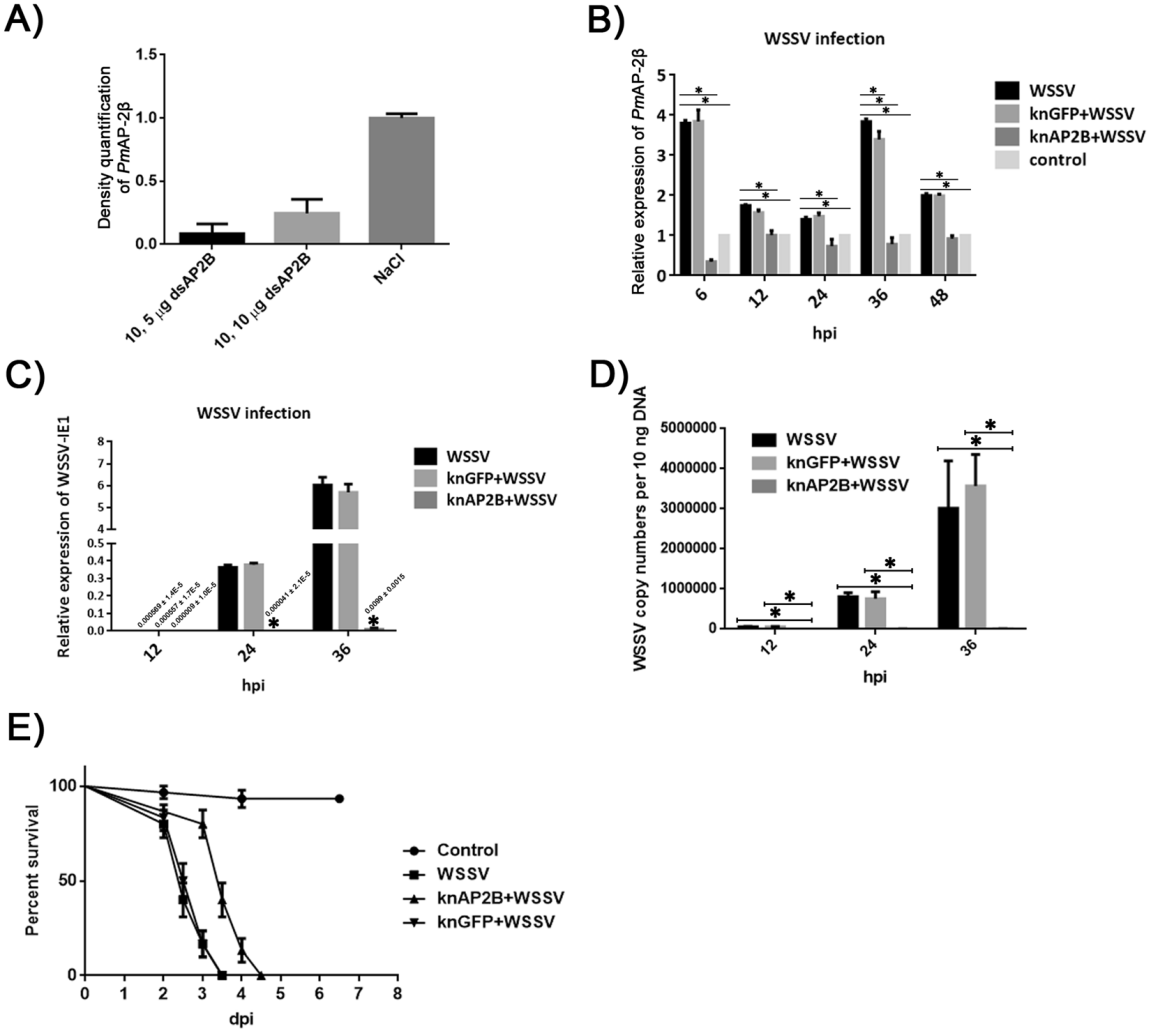
Effect of *Pm*AP-2β knockdown during WSSV infection. (**A**) Determination of *Pm*AP-2β transcript level in *P*. *monodon* hemocytes after RNAi-mediated gene silencing. Shrimp were double injected with either 10 and 5 μg *Pm*AP-2β dsRNA per 1 g of shrimp or 10 and 5 μg *Pm*AP-2β dsRNA per 1 g of shrimp or NaCl. The *Pm*AP-2β transcript level was measured by Quantitative Real-time RT-PCR and normalized to that of EF1-α gene (an internal control). (**B**) Expression level of *Pm*AP-2β in unchallenged, WSSV-challenged, WSSV-challenged + GFP knockdown and WSSV-challenged + *Pm*AP-2β knockdown shrimp at 6, 12, 24, 36, 48 hpi. Quantitative Real-time RT-PCR was performed to analyze *Pm*AP-2β transcript levels. (**C**) Determination of WSSV IE1 transcript level in WSSV-challenged, WSSV-challenged + GFP knockdown and WSSV-challenged + *Pm*AP-2β knockdown shrimp at 12, 24 and 36 hpi. The mRNA expression levels of WSSV IE1 were determined by Quantitative Real-time RT-PCR. (**D**) Analysis of WSSV copy number in WSSV-challenged, WSSV-challenged + GFP knockdown and WSSV-challenged + *Pm*AP-2β knockdown shrimp at 12, 24 and 36 hpi. WSSV copy number was determined as described in Jaturontakul *et al*.^54^. (**E**) Cumulative mortality of shrimp after challenged with WSSV. Shrimp were divided into 4 groups (control, WSSV-challenged, WSSV-challenged + GFP knockdown and WSSV-challenged + *Pm*AP-2β silenced shrimp) with 12 individuals per group. The data are shown as the mean ± standard deviation. An asterisk indicates significant differences from control group (*p* < 0.05). All experiments were carried out in triplicates.

An appropriate amount of *Pm*AP-2β dsRNA to be used in gene silencing experiments was examined in *P*. *monodon*. Shrimp were doubly injected with 10 µg *Pm*AP-2β dsRNA per 1 g shrimp and either 5 or 10 µg *Pm*AP-2β dsRNA per 1 g shrimp at 24 h interval. Hemocytes were collected at 24 h after second injection and 10, 5 µg *Pm*AP-2β dsRNA injection seemed to show stronger effect on silencing *Pm*AP-2β expression than 10, 10 µg *Pm*AP-2β dsRNA injection (Fig. 5A). As a result, 10, 5 µg *Pm*AP-2β dsRNA injection was used in RNAi experiments. As shown in Fig. 5B, *Pm*AP-2β was significantly up-regulated during WSSV infection and shrimp injected with GFP dsRNA possessed similar level of *Pm*AP-2β transcript to that of normal shrimp challenged by WSSV. Meanwhile, shrimp repeatedly injected with 10, 5 µg *Pm*AP-2β dsRNA and WSSV showed dramatically lower level of *Pm*AP-2β transcripts at 6, 12, 24, 36 and 48 hpi, in comparison with those in WSSV-challenged and WSSV-challenged + GFP knockdown shrimp. This result indicated that *Pm*AP-2β was specifically knockdown throughout the experiments. *Pm*AP-2β silenced shrimp exhibited a significantly lower level of WSSV IE-1 transcripts at 24 and 36 hpi, compared with those in WSSV-challenged and WSSV-challenged + GFP knockdown shrimp (Fig. 5C). In addition, *Pm*AP-2β silenced shrimp had a significant lower viral copy numbers at 12, 24 and 36 hpi than that of WSSV-challenged and WSSV-challenged + GFP knockdown shrimp (Fig. 5D). These suggested that *Pm*AP-2β silencing affected WSSV propagation. Similar conclusions were drawn from mortality assay, in which delayed mortality of shrimp was observed in *Pm*AP-2β knockdown group (Fig. 5E). Clearly, *Pm*AP-2β is involved in WSSV infection.

### *Pm*AP-2β localized with WSSV during viral infection

**Figure 6:**
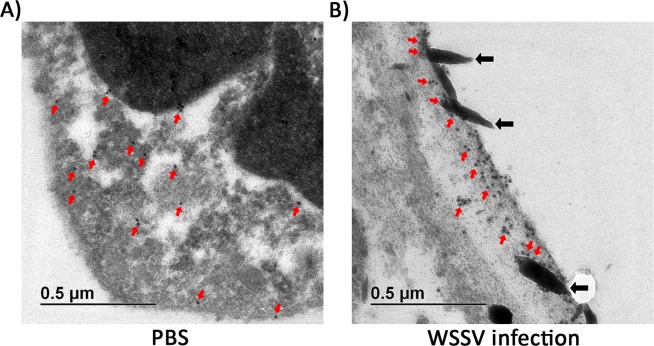
Localization of *Pm*AP-2β in uninfected (**A**) and WSSV infected (**B**) hemocyte cells by immunoelectron microscopy. *Pm*AP-2β was detected using 10 nm gold-conjugated anti-AP-2β antibody. Red arrows indicate gold particles and black arrows show WSSV particles.

In unchallenged hemocytes, immuno-gold particles, representing *Pm*AP-2β, were presented in a wide area of cytoplasm (red arrows) (Fig. 6A). On the contrary, *Pm*AP-2β was mostly accumulated at the plasma membrane of WSSV-challenged shrimp hemocytes and the internalized WSSV particles were surrounded by *Pm*AP-2β proteins (black arrows) (Fig. 6B). TEM visualization of *Pm*AP-2β and viral particles confirmed that WSSV enters shrimp cells via clathrin-mediated endocytosis.

### Effects of *Pm*AP-2β knockdowns on signaling pathways and immune responses during WSSV infection

**Figure 7:**
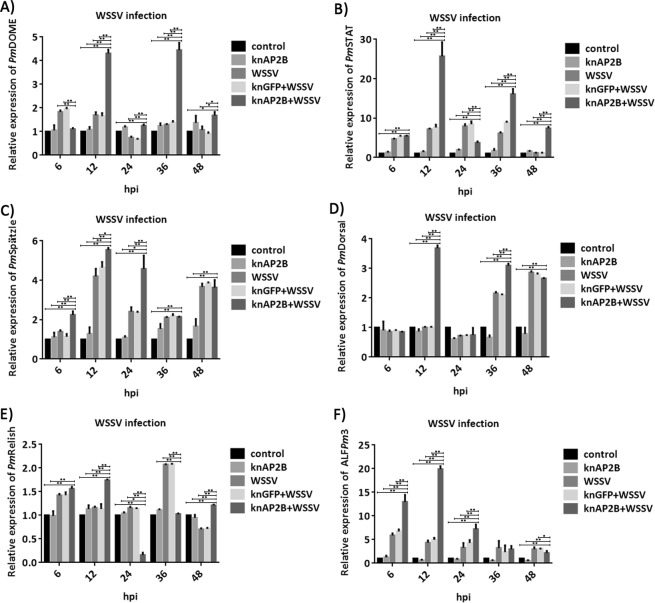
Relative transcription levels of immune related genes in *Pm*AP-2β silenced shirmp during WSSV infection. Shrimp hemocytes were collected for qRT-PCR analysis of *Pm*DOME (**A**), *Pm*STAT (**B**), *Pm*Spätzle (**C**), *Pm*Dorsal (**D**), *Pm*Relish (**E**) and ALF*Pm*3 transcript levels at 6, 12, 24, 36 and 48 hpi. The immune relate genes expression in each groups were calculated relative to that of the control. Data are presented as mean ± standard deviations. An asterisk (*) and double-asterisk (**) represents significant differences from control group (*p* < 0.05) and (*p* < 0.01), respectively.

WSSV entry may trigger several signaling pathways, including Toll, Imd and JAK/STAT pathways, as well as other immune related genes such as antimicrobial peptides. In this work, the gene expression of *Pm*DOME, *Pm*STAT, *Pm*Spätzle, *Pm*Dorsal, *Pm*Relish and ALF*Pm*3 were determined by qRT-PCR. *Pm*DOME and *Pm*STAT present in the JAK/STAT signaling pathway, while *Pm*Spätzle and *Pm*Dorsal are in the Toll pathway and *Pm*Relish belongs to the Imd pathway. ALF*Pm*3 was reported to reduce WSSV propagation^36^ and interact with several WSSV structural proteins^37^. During WSSV infection, levels of *Pm*DOME and *Pm*STAT transcripts in *Pm*AP-2β silenced shrimp were significantly higher than that in normal shrimp infected with WSSV (Fig. 7A,B). Especially at 12 hpi, *Pm*DOME and *Pm*STAT were up-regulated by 4.3-fold and 25.7-fold, respectively. *Pm*AP-2β silencing increased expression of *Pm*Spätzle at 6, 12, 24 hpi and *Pm*Dorsal transcription level at 12 and 36 h post-WSSV infection (Fig. 7C,D). The expression of *Pm*Relish was highest at 36 hpi and increased by 2.1-fold, in comparison to uninfected shrimp (Fig. 7E). The *Pm*Relish transcript levels of WSSV-challenged *Pm*AP-2β knockdown shrimp were increased at 12 and 48 hpi, but significantly decreased at 24 and 36 hpi, when compared with normal shrimp infected with WSSV. Overall, the data suggested that *Pm*AP-2β may be related to the JAK/STAT and the Toll pathways, but not the Imd pathway. During WSSV infection, ALF*Pm*3 was up-regulated by 5.9-, 4.4- and 3.3-fold at 6, 12 and 24 hpi (Fig. 7F). Surprisingly, expression of ALF*Pm*3 was significantly enhanced in *Pm*AP-2β silenced shrimp by 13-, 20- and 7.2-fold at 6, 12 and 24 hpi, respectively.

## Discussion

This work aims to characterize the clathrin adaptor proteins from *P*. *monodon*, *Pm*AP-2β and *Pm*AP-2μ and study clathrin-mediated endocytosis during WSSV infection. Previously, *Pm*-clathrin coat AP17 or *Pm*AP-2σ has been characterized^22^. Immunofluorescence confocal microscopy showed that level of *Pm*-clathrin coat AP17 was increased during WSSV infection and *Pm*-clathrin coat AP17 co-localized with WSV477, a Cys2/Cys2-type zinc finger regulatory protein with ATP/GTP-binding activity, in the cytoplasm of shrimp hemocytes (Fig. 1A). It is likely that *Pm*-clathrin coat AP17 was up-regulated in response to WSSV infection. In a previous report, silencing WSV477 resulted in a reduction of viral late gene VP28 transcript. In addition, WSV477 was reported to bind to a Kazal serine proteinase inhibitor SPI*Pm*2^38^.

*Pm*-clathrin coat AP17 gene silencing and CPZ treatment resulted in a lower expression of WSSV-IE1 (Fig. 1B). This indicated that clathrin-mediated endocytosis is involved in WSSV infection in *P*. *monodon*. CPZ treatment showed stronger inhibitory effects on WSSV infection than that of *Pm*-clathrin coat AP17 gene knockdown. *Pm-*clathrin coat AP17 gene silencing interferes with the assembly of clathrin and the formation of coated pits on the plasma membrane, while CPZ inhibits clathrin coated vesicles from recycling.

The complete *Pm*AP-2β and *Pm*AP-2μ cDNA sequences were reported and the amino acid sequences were analyzed. AP-2β and AP-2μ are highly conserved across diverse phyla. In addition, the recombinant *Pm*AP-2β and *Pm*AP-2μ were successfully expressed and purified as shown in Fig. S1 in Supplementary Information. An apparent molecular weight of *Pm*AP-2β and *Pm*AP-2μ determined by SDS-PAGE was in a good agreement with the calculated molecular mass.

*Pm*AP-2β was up-regulated in response to WSSV infection (Fig. 4C). At 6 and 36 hpi, the *Pm*AP-2β transcript levels in WSSV-infected shrimp were increased by 4–fold, compared with uninfected shrimp. *Pm*AP-2β silenced shrimp showed significantly lower WSSV-IE1 mRNA level than that in normal shrimp infected with WSSV (Fig. 5C). It was previously shown that AP-2 is important for endocytic coated-pit and coated-vesicle formation at the plasma membrane and very few clathrin coats were observed after AP-2 knockdown in BSC1 and HeLa cells^39^. Disruption of clathrin-dependent trafficking by *Pm*AP-2β silencing resulted in a reduction of WSSV copy number (Fig. 5D). This was in agreement with the cumulative mortality experiment showing that *Pm*AP-2β silencing decreased mortality rates (Fig. 5E). TEM demonstrated that *Pm*AP-2β accumulated at plasma membrane and facilitated WSSV entry (Fig. 6B). To our knowledge, this is the first report showing clear vision of WSSV internalized into the host cells via clathrin-dependent endocytosis.

Our results confirmed that WSSV internalizes via clathrin-mediated endocytosis. Similar findings were reported in crayfish hematopoietic tissues^13,14^ and the pacific white shrimp *L*. *vannamei*^12^. Huang, Z. *et al*. 2013 suggested that WSSV use the caveolae-mediated endocytosis to enter the shrimp cells^40^. Chen, R. *et al*. 2016 showed that WSSV entry into hematopoietic tissues of the crayfish is dependent on multiple endocytic routes, including clathrin-mediated endocytosis, macropinocytosis and caveolae-dependent endocytosis^14^. *Pm*AP-2β knockdown resulted in a delayed mortality, but did not completely inhibit WSSV infection. This suggested that WSSV may enter shrimp cells via multiple routes.

Relative transcription levels of immune related genes in *Pm*AP-2β silenced shrimp were compared with those in normal shrimp during WSSV infection (Fig. 7). *Pm*AP-2β silencing resulted in up-regulation of *Pm*DOME, *Pm*STAT, *Pm*Spätzle and *Pm*Dorsal during WSSV infection. It is possible that *Pm*AP-2β is somehow associated with the JAK/STAT and the Toll pathway. In contrast, effect of *Pm*AP-2β knockdown on *Pm*Relish expression level was less evident, suggesting that *Pm*AP-2β may not involve in the Imd pathway.

ALF*Pm*3 was up-regulated in response to WSSV infection. *Pm*AP-2β silencing enhanced ALF*Pm*3 expression at the highest level by 20-fold at 12 h after WSSV challenge (Fig. 7F). It was shown previously that ALF*Pm*3 exhibited anti-WSSV activity via binding to the viral structural proteins, resulting in diminishing of WSSV virions^37,41^. A significant increase of ALF*Pm*3 expression may cause a delayed mortality (Fig. 5E).

Transcriptomic and comparative proteomic data have shown that shrimp Toll receptors play a role during WSSV infection^42,43^. An RNAi study of a Toll receptor from giant freshwater prawn *Macrobrachium rosenbergii* (*Mr*Toll) showed that the ALF genes (ALF2, ALF3, ALF4 and ALF5) were regulated by *Mr*Toll during WSSV challenge^44^. Li, H. *et al*., 2018 reported that Toll4 from *L*. *vannamei* inhibited WSSV infection through activation of Dorsal to induce antimicrobial peptides, including ALF and lysozyme^45^. Injection of *Pm*Spätzle 1 into a normal shrimp increased ALF*Pm*3, crustin*Pm*1, crustin*Pm*7 and penaeidin3 synthesis^46^. Based on these evidences, it is possible that *Pm*AP-2β silencing may activate the Toll pathway through *Pm*Dorsal, and possibly *Pm*Spätzle, leading to a significant increase of ALF*Pm*3 transcription during WSSV infection (Fig. 7).

In Drosophila, recruitment and trafficking of the clathrin-AP complexes into endocytic vesicles towards the lysosome is required to activate JAK/STAT signaling^47^. In *L*. *vannamei*, silencing STAT decreased shrimp mortality caused by WSSV and specific inhibitor of STAT3 (S3I-201) inhibited WSSV replication in hematopoietic tissue of crayfish *C*. *quadricarinatus*^48^. These findings seemed to contradict our results, whereby *Pm*AP-2β silencing, which may interrupt clathrin-vesicle formation and trafficking, resulted in activation of *Pm*DOME and *Pm*STAT in the JAK/STAT pathway (Fig. 7A,B). During WSSV infection, *Pm*STAT in *Pm*AP-2β silenced shrimp was up-regulated by 25.7–fold at 12 hpi (Fig. 7B). *Pm*AP-2β knockdown shrimp also had a reduced mortality rate caused by WSSV (Fig. 5E). Based on our results, we hypothesized that *Pm*AP-2β silencing may trigger the JAK/STAT pathway, leading to anti-WSSV response. *Lv*JAK silencing caused higher mortality^49^ and knockdown of *Lv*SOC2, a negative feedback loop regulator of JAK/STAT pathway in *L*. *vannamei*, resulted in less WSSV infection^50^. More recently, Wang, W. *et al*., 2019 reported that inhibition of the JAK/STAT pathway in Drosophila by WSV181 could enhance WSSV replication^51^. These findings indicated that the JAK/STAT pathway could function as anti-viral response. Role of clathrin-AP2 on the JAK/STAT pathway need to be further investigated.

When clathrin-dependent endocytosis is interrupted, WSSV may enter shrimp cells via alternative routes e.g. caveolae-dependent endocytosis and macropinocytosis^14^. Caveolae are cholesterol- and sphingolipid-rich smooth invaginations of the plasma membrane that mediate internalization of various molecules. Caveolae are referred as clathrin-independent endocytosis and contain caveolins as the main structural proteins^52^. Caveolin-1 was reported to inhibit STAT5a activation by direct interaction with JAK2^53^. Caveolin proteins possess the caveolin-scaffolding domain, which is similar to the SOCS kinase inhibitory region (KIR) domain, so caveolin might function as negative regulators of receptor signaling pathway via JAK/STAT.

In conclusion, this work has confirmed that WSSV enter shrimp cells via clathrin-mediated endocytosis. The complete sequences of a full-length cDNA for *Pm*AP-2β and *Pm*AP-2μ were reported and the recombinant proteins were expressed and purified. Silencing of clathrin AP-2 components, endocytosis inhibitor, immunofluorescence confocal microscopy and TEM suggested that clathrin-mediated endocytosis play an important role during WSSV infection. In addition, *Pm*AP-2β may be related to the JAK/STAT and the Toll pathways in response to WSSV.

## Methods

### Shrimp

Pathogen-free black tiger shrimp, *P*. *monodon*, of about 3.5 ± 0.1 g bodyweight were obtained from Charoen Pokphand Farm in Chanthaburi Province, Thailand. The animals were kept in aerated water within 120 L laboratory tanks at temperature of 28 ± 4 °C and a salinity of 20 ppt for at least 1 week prior to the experiments.

### Preparation of WSSV stock

WSSV was prepared from the gill tissue of WSSV-infected moribund shrimp using centrifugation and membrane filtration as described in Jaturontakul *et al*.^54^.

### Total RNA isolation and first-stranded cDNA synthesis

Shrimp tissues were collected and homogenized in FARB buffer (Tissue Total RNA mini kit, Favorgen, Taiwan). Total RNA was extracted and the first-strand cDNA was synthesised by First-strand cDNA Synthesis Kit (Thermo Fisher, USA). The cDNA was kept at −20 °C until further experiments.

### Effect of clathrin coat AP17 gene silencing and chlorpromazine (CPZ) pre-treatment on WSSV propagation in shrimp

*P*. *monodon* were divided into four groups and each group contained nine shrimp. In group 1, shrimp were injected with 150 mM NaCl, while in group 2 and 3, shrimp were injected with 10 µg of GFP dsRNA per 1 g of shrimp and 10 µg of clathrin coat AP17 dsRNA per 1 g of shrimp, respectively. Shrimp in group 4 were given 0.25 µg CPZ per 1 g of shrimp. After 24 h, WSSV (~6 × 10^6^ viral copies) were mixed with either 150 mM NaCl, 10 µg of GFP dsRNA per 1 g of shrimp or 10 µg of *Pm*-clathrin coat AP17 dsRNA per 1 g of shrimp and injected into shrimp. At 12 h post injection, hemolymph was collected for total RNA extraction and cDNA synthesis. To determine the intermediate early gene of WSSV IE-1 transcript levels, quantitative Real-time RT-PCR was carried out as 95 °C for 30 s, 40 cycles of 95 °C for 5 s and 55 °C for 10 s, using specific primers for WSSV-IE1 (See Supplementary Information, Table S1). Elongation factor-1 alpha (EF-1α) gene was used as an internal control. The experiment was performed in triplicates. Mathematical model was used to analyze the threshold cycle (C_T_)^55^. Statistical analysis was performed using the one-way ANOVA followed by post hoc test. The result differences were considered significant at *p* < 0.05 (*), *p* < 0.01 (**).

Comparative *C*_T_ method was used to compare the gene expression in two different samples. The fold change of gene expression was calculated using the following formula.

$${\rm{Fold}}\,{\rm{change}}={{\rm{2}}}^{-\Delta \Delta \mathrm{CT}}$$

$$\Delta \Delta {C}_{T}=[{(C}_{{\rm{T}}}{\rm{gene}}\,{\rm{of}}\,{\rm{interest}}-{{C}}_{{\rm{T}}}{\rm{internal}}\,{\rm{control}}){\rm{sample}}\,{\rm{A}}-({{C}}_{{\rm{T}}}\,{\rm{gene}}\,\mathrm{of}\,\,{\rm{interest}}-{{C}}_{{\rm{T}}}{\rm{internal}}\,{\rm{control}}){\rm{sample}}\,{\rm{B}})]$$

### Immunofluorescence and confocal microscopy

A procedure used to detect *Pm*-clathrin coat AP17 by immunofluorescence and confocal microscopy was previously describe in Jatuyosporn *et al*.^22^. Either WSSV solution or 150 mM NaCl was injected into shrimp and hemolymph was collected from three individual shrimp at 12 h post-injection, pooled and mixed in equal volume of 4% paraformaldehyde in PBS. Hemocytes were collected by centrifugation (800 × g for 10 min at 4 °C), washed 3 times with PBS and fixed on microscope slides. Hemocytes were then incubated with 0.1% Triton X-100 in PBS for 5 min and washed triplicate with PBS. *Pm*-clathrin coat AP17 was probed using purified rabbit anti-*Pm*-clathrin coat AP17 polyclonal IgG antibody^22^ diluted 1:50 in PBSF (PBS with 1% (v/v) FBS), followed by a 1:500 dilution of Alexa Fluor 488 goat anti-rabbit IgG antibody (Invitrogen). WSSV was detected by polyclonal IgG antibody specific to WSV477^38^, diluted 1:50 in PBSF, followed by a 1:1000 dilution of Alexa Fluor 568 goat anti-mouse IgG antibody (Invitrogen). Nuclei were stained with 1:1500 dilution of TOPRO-3 (Invitrogen) in PBS. The microscope slides containing the stained and fixed hemocytes were coated by ProLong Gold (Invitrogen) and stored in the dark at 4 °C until they were examined by a confocal fluorescence microscopy.

### Gene cloning, sequence alignment and phylogenetic analysis of *Pm*AP-2β and *Pm*AP-2µ

The complete sequence of a full length cDNA for *Pm*AP-2β and the partial cDNA sequence for *Pm*AP-2µ were obtained from the *P*. *monodon* EST database (http://pmonodon.biotec.or.th). 5′-Rapid Amplification of the cDNA Ends (RACE) was carried out using SMARTer RACE 5′/3′ kit (Takara, USA) and specific primers as shown in Table S1. In brief, 1 μg of total RNA was used to generate 5′-RACE cDNA libraries, of which 25 μl were diluted by the addition of 90 µl of Tricine-EDTA and 1 μl of the solution was used as a template in RACE reaction. DNA fragment of *Pm*AP-2µ was amplified by specific RACE primer (Table S1) with the following conditions: 25 cycles of 94 °C for 30 s, 68 °C for 30 s and 72 °C for 3 min. The PCR product was then purified and ligated to linearized pRACE vector. Ligation mixture was transformed into Stellar competent cell. The plasmid DNA was extracted from the positive colony for sequencing (Macrogen).

The full length *Pm*AP-2β and *Pm*AP-2µ genes were amplified from cDNA of normal *P*. *monodon*, using PCR condition as followed: 98 °C for 3 min (denaturation), followed by 30 cycles of 98 °C for 30 s, 60 °C for 30 s and 72 °C for 90 s (for *Pm*AP-2β) or 60 s (for *Pm*AP-2µ) and a final extension at 72 °C for 2 min. The specific primers for amplification of *Pm*AP-2β and *Pm*AP-2µ were designed to contain 5′ *EcoR* I and 3′ *Xho* I restriction sites for *Pm*AP-2β and 5′ *Nco* I and 3′ *Xho* I restriction sites for *Pm*AP-2µ (Table S1). The amplified full-length *Pm*AP-2β (~2.8 kb) and *Pm*AP-2µ (~1.3 kb) DNA fragments were ligated with pET-28b(+) expression vector (Novagen). The ligation mixture was then transformed into *Escherichia coli* TOP10 (Invitrogen). The recombinant plasmids, pET28b-*Pm*AP-2β and pET28b-*Pm*AP-2µ, were verified by sequencing.

The polyadenylation site in *Pm*AP-2β and *Pm*AP-2μ sequences was predicted by Poly(A) Signal Miner (http://dnafsminer.bic.nus.edu.sg/PolyA.html)^56^ and the predicted molecular weight of *Pm*AP-2β and *Pm*AP-2μ was calculated by Compute pI/MW (https://web.expasy.org/compute_pi)^57^. Bioinformatics analysis of *Pm*AP-2β and *Pm*AP-2µ was carried out using the basic local alignment search tool (BlastX, http://blast.ncbi.nlm.nih.gov/)^58^, multiple sequence alignment (Clustal Omega, https://www.ebi.ac.uk/Tools/msa/clustalo/)^59^ and phylogenetic tree (iTOL, https://itol.embl.de/)^60^. The secondary structure and protein domain of *Pm*AP-2β and *Pm*AP-2μ were predicted by Jpred4 (http://www.compbio.dundee.ac.uk/jpred/)^61^, PROSITE (http://prosite.expasy.org)^62^ and InterPro (https://www.ebi.ac.uk/interpro/)^63^.

### Expression and purification of the recombinant *Pm*AP-2β and *Pm*AP-2µ

*E*. *coli* strain BL21 (DE3) CodonPlus-RIL (Stratagene) harboring either the recombinant plasmid pET28b-*Pm*AP-2β or pET28b-*Pm*AP-2µ was grown in LB broth containing 34 μg/ml kanamycin at 37 °C until OD_600_ reached 0.4. Then, isopropyl-β-D-thio-galactoside (IPTG) with a final concentration of 1 mM was added into the media and incubated for 4 h before the bacterial cells were harvested and resuspended in 1x PBS buffer, pH 7.4. Cells were disrupted by sonication and inclusion bodies were collected by centrifugation at 5000 xg for 10 min at 4 °C. A denaturing solution containing 1x PBS, pH 7.4, 8 M urea and 1% Triton X-100 was used to solubilized the inclusion bodies. The recombinant *Pm*AP-2β and *Pm*AP-2µ were purified by a Ni Sepharose 6 Fast Flow column (GE Healthcare) under denaturing conditions and eluted stepwise with a denaturing solution containing 300 mM imidazole. Protein fractions were then analyzed by 10% sodium dodecyl sulphate polyacrylamide gel electrophoresis (SDS–PAGE). The purified protein was then dialyzed against 1x PBS buffer pH 7.4, containing 4, 2 and 0 M urea. After removing any remaining precipitant by centrifugation, the renatured protein was analyzed by Western blotting using a primary anti-His antibody (GE Healthcare). The concentration of purified recombinant proteins were determined by bicinchoninic acid (BCA) protein assay (Pierce).

### Analysis of *Pm*AP-2β gene expression in shrimp

Shrimp tissues, including hemocyte, eyestalk, epipodite, gill, heart, lymphoid organ, stomach, intestine, hepatopancreas and muscle, were collected from 3 normal shrimp and total RNA was extracted by Tissue Total RNA mini kit (Favorgen), followed by cDNA synthesis using the RevertAid First Strand cDNA Synthesis Kit (Thermo Fisher). The *Pm*AP-2β gene expression level in each tissue was identified by RT-PCR using 1 µl of cDNA template and *Pm*AP-2β (RT-PCR) primers shown in Table S1. Elongation factor 1 α (EF-1α) gene was used as an internal control. The PCR reaction was initiated by 94 °C for 3 min, followed by 30 cycles of 94 °C for 30 s, 55 °C for 30 s and 72 °C for 30 s and a final extension at 72 °C for 10 min. The PCR products were then analyzed by 2% (w/v) agarose gel electrophoresis.

### Expression level of *Pm*AP-2β mRNA in unchallenged and WSSV challenged shrimp hemocytes

Shrimp were injected with either ~6 × 10^6^ viral copies of WSSV (challenged-shrimp) or 150 mM NaCl (unchallenged-shrimp); and the hemocytes were then collected from both groups (3 shrimp per group) at 6, 12, 24, 36 and 48 h post-injection using 500 µl of modified Alsever solution (MAS: 27 mM sodium citrate, 336 mM NaCl, 115 mM glucose, 9 mM EDTA, pH 7.0), per 500 µl of hemolymph. Total RNA extraction was performed, followed by cDNA synthesis as previously described. Real-time RT-PCR was carried out using an equal amount of cDNAs in iCycler iQTM Real-Time detection system and the SsoFast EvaGreen Supermix (Bio-Rad) in the following conditions: one cycle at 95 °C for 30 s, followed by 40 cycles of 95 °C for 5 s and 55 °C for 10 s, using *Pm*AP-2β and EF-1α (real-time PCR) primers as shown in Table S1. The experiment was carried out in triplicates. Comparative C_T_ method was used to compare the gene expression in WSSV challenged-(sample A) and unchallenged (sample B) shrimp^64^ and statistical analysis was performed using the one-way ANOVA followed by post hoc test (Duncan’s new multiple range test).

### RNAi-mediated *Pm*AP-2β gene silencing and quantitative determination of *Pm*AP-2β transcript levels and WSSV copy numbers

The recombinant *Pm*AP-2β and GFP plasmids were used as templates to amplify *Pm*AP-2β dsRNA and GFP dsRNA, respectively. As shown in Table S1, a pair of primers specific to either *Pm*AP-2β or GFP were designed, one of which contained the T7 promoter sequence at 5′ (double-underlined). The two PCR products (*Pm*AP-2β and GFP) were separately amplified by specific primer pairs with the following conditions: 94 °C for 3 min, followed by 40 cycles of 94 °C for 30 s, 55 °C for 30 s and 72 °C for 30 s and a final extension at 72 °C for 10 min. The two PCR product templates were *in vitro* transcribed using the T7 RiboMAX System (Promega) to produce two complementary single-stranded RNAs. After that, RQ1 RNase- free DNase was added and incubated at 37 °C for 1 h to remove the DNA template, followed by phenol–chloroform extraction. Double-stranded RNA was obtained by mixing equal amounts of each of the complementary single-stranded RNAs, incubated at 70 °C for 10 min, and slowly cooled down at room temperature. *Pm*AP-2β dsRNA and GFP dsRNA were analyzed by 1% agarose gel electrophoresis and concentrations of newly synthesized dsRNAs were determined by measuring absorbance at 260 nm.

Shrimp of approximately 3 g body weight were divided into four groups with three shrimp per group. Shrimp in the first control group (group 1) and the second control (group 2) were injected with either 150 mM NaCl or 10 µg of GFP dsRNA per 1 g of shrimp, respectively, while shrimp in gene knockdown groups were injected with either 5 or 10 µg of *Pm*AP-2β dsRNA per 1 g of shrimp (group 3 and 4, respectively). The injection was repeated at 24 h after the first injection; and hemolymph was collected from each shrimp at further 24 h. Total RNA extraction and cDNA synthesis were carried out as previously described. RT-PCR was performed in order to evaluate the degree of gene transcript silencing using the *Pm*AP-2β and EF-1α primers (Table S1). The PCR product was analyzed by 2% (w/v) agarose gel electrophoresis, and *Pm*AP-2β gene expression level was reported as relative to that of EF-1α.

WSSV IE-1 transcript levels in WSSV-challenged, WSSV-challenged-knGFP and WSSV-challenged-*Pm*AP-2β silenced shrimp were measured by quantitative Real-time RT-PCR as described above.

To determined WSSV copy numbers in WSSV-challenged, WSSV-challenged-knGFP and WSSV-challenged-*Pm*AP-2β silenced shrimp, the total DNA was extracted from shrimp hemocytes and Real-time PCR was performed as described in Jaturontakul *et al*.^54^.

### Mortality assay of *Pm*AP-2β silencing shrimp upon WSSV infection

*P*. *monodon* were separated into four groups with 12 individuals per group. Shrimp in the first control groups (group 1 and 2) and the second control group (group 3) were injected with either 150 mM NaCl (group 1 and 2) or 10 µg of GFP dsRNA per 1 g of shrimp (group 3). Meanwhile, shrimp in gene knockdown group (group 4) were injected with 10 µg of *Pm*AP-2β dsRNA per 1 g of shrimp. After 24 h, WSSV (~6 × 10^6^ viral copies) were mixed with either 150 mM NaCl, 10 µg of GFP dsRNA per 1 g of shrimp or 10 µg of *Pm*AP-2β dsRNA per 1 g of shrimp and injected into shrimp. The shrimp in control group (group 1) were injected with 150 mM NaCl instead of WSSV. The mortality was recorded every 12 hpi up to 7 days. This experiment was performed in triplicate. Data were analyzed using GraphPad Prism 6 plot, and presented as percent survival with the *p* values calculated by logrank test.

### Localization of *Pm*AP-2β in unchallenged and WSSV challenged shrimp

*P*. *monodon* gills were dissected from unchallenged and WSSV challenged shrimp at 12 hpi. Dissected gill was fixed in 4% paraformaldehyde in PBS for 30 min on ice. Then, gills were washed twice by ice-cold PBS and prepared embedding with LR White Embedding Medium (EMS) according to manufacturer’s protocol. The embedded tissues were cut into ultrathin section (60–70 nm) and placed on Formvar-supported and carbon-coated nickel grid. The primary AP-2β antibody (abcam) was conjugated with gold nano particles using InnovaCoat Gold Conjugation kit according to manufacturer’s protocol. The conjugated antibody was diluted 1:50 in an incubation buffer (1% BSA in PBS). The grids were incubated with blocking buffer (1% BSA and 5% normal Serum in PBS) for 30 min and then incubated with a diluted conjugated antibody at 4 °C overnight. After several washes with PBS and MilliQ water, the grids were stained with uranyl acetate solution for 5 min, followed by Reynols lead citrate solutions for 2 min and observed under transmission electron microscope.

### Immune related gene expression in *Pm*AP-2β silenced shrimp during WSSV infection

Shrimp were divided into four control groups (group 1–4) and one experimental group (group 5). Shrimp in group 1 and 2 were injected with 150 mM NaCl, while shrimp in group 3 and group 4 were injected with either 10 µg of *Pm*AP-2β dsRNA per 1 g of shrimp or 10 µg of GFP dsRNA per 1 g of shrimp, respectively. Shrimp in group 5 were also injected with 10 µg of *Pm*AP-2β dsRNA per 1 g of shrimp. Double injection was performed at 24 h intervals. At 24 h after the second injection, WSSV (~6 × 10^6^ viral copies) were mixed with either 150 mM NaCl, 10 µg of GFP dsRNA per 1 g of shrimp or 10 µg of *Pm*AP-2β dsRNA per 1 g of shrimp and injected into shrimp. Shrimp in control group 1 and 3 were injected with 150 mM NaCl instead of WSSV. Hemolymph was collected from each shrimp at 6, 12, 24, 36 and 48 hpi. Total RNA extraction and cDNA synthesis were performed as described above. The qRT-PCR was carried out using specific primers of immune related genes as shown in Table S1 and the qRT-PCR condition was 95 °C for 30 s, followed by 40 cycles of 95 °C for 5 s and 55 °C for 10 s. The experiment was carried out in triplicates. Comparative C_T_ method was used to compare the immune related gene expression in two different samples, WSSV challenged normal-(sample A) and WSSV challenged *Pm*AP-2β knockdown (sample B) shrimp. Statistical analysis was done using the one-way ANOVA followed by post hoc test (Duncan’s new multiple range test). The result differences were considered significant at *p* < 0.05 (*) and *p* < 0.01 (**).

## Supplementary information

Role of Clathrin Assembly Protein-2 Beta Subunit during White Spot Syndrome Virus Infection in Black Tiger Shrimp Penaeus monodon
