# Supplementary material for: Role of Clathrin Assembly Protein-2 Beta Subunit during White Spot Syndrome Virus Infection in Black Tiger Shrimp Penaeus monodon
Source: Sci Rep. 2019 Sep 17;9:13489. doi: 10.1038/s41598-019-49852-0 (PMC6748913; doi:10.1038/s41598-019-49852-0)
Supplement: Supplementary file 1 — Role of Clathrin Assembly Protein-2 Beta Subunit during White Spot Syndrome Virus Infection in Black Tiger Shrimp Penaeus monodon [file 41598_2019_49852_MOESM1_ESM.docx]

***Role of Clathrin Assembly Protein-2 Beta Subunit during White Spot Syndrome Virus Infection in Black Tiger Shrimp Penaeus monodon***

Thapanan Jatuyosporn^1,2^, Pasunee Laohawutthichai^1,2^, Premruethai Supungul^3^, Rogerio R. Sotelo-Mundo^4^, Adrian Ochoa-Leyva^5^, Anchalee Tassanakajon^2^ and Kuakarun Krusong^1*^

^1^ Structural and Computational Biology Research Unit, Department of Biochemistry, Faculty of Science, Chulalongkorn University, Bangkok 10330, Thailand

^2^ Center of Excellence for Molecular Biology and Genomics of Shrimp, Department of Biochemistry, Faculty of Science, Chulalongkorn University, Bangkok 10330, Thailand

^3^ National Center for Genetic Engineering and Biotechnology (BIOTEC),

National Science and Technology Development Agency (NSTDA), Pathumthani 12120, Thailand

^4^ Laboratorio de Estructura Biomolecular, Centro de Investigación en Alimentación y Desarrollo, A.C. (CIAD). Carretera Gustavo Enrique Astiazaran Rosas No. 46, Hermosillo, Sonora 83304, Mexico

^5^ Departamentos de Microbiología Molecular, Medicina Molecular y Bioprocesos, Unidad Universitaria de Secuenciacián Masiva y Bioinformática, Instituto de Biotecnología (IBT), Universidad Nacional Autónoma de México (UNAM), Avenida Universidad 2001, Colonia Chamilpa, Cuernavaca 62210, Mexico

^*^ To whom correspondence should be addressed:

Kuakarun Krusong, Ph.D.

Department of Biochemistry, Faculty of Science,

Chulalongkorn University, Bangkok 10330, Thailand

Tel: +66 (0)2 218 5413

Email: Kuakarun.K@chula.ac.th

**Supplementary Information**


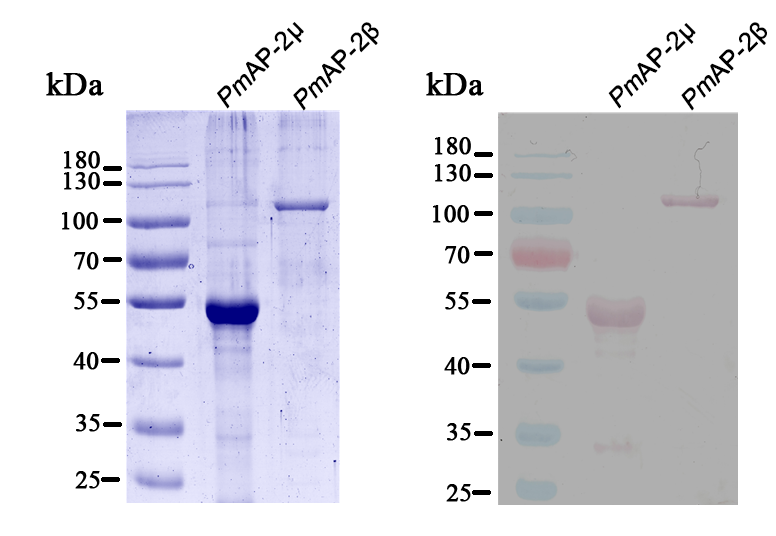


**Fig. S1** SDS-PAGE (A) and Western blot analysis (B) of purified recombinant AP-2β and AP-2μ.


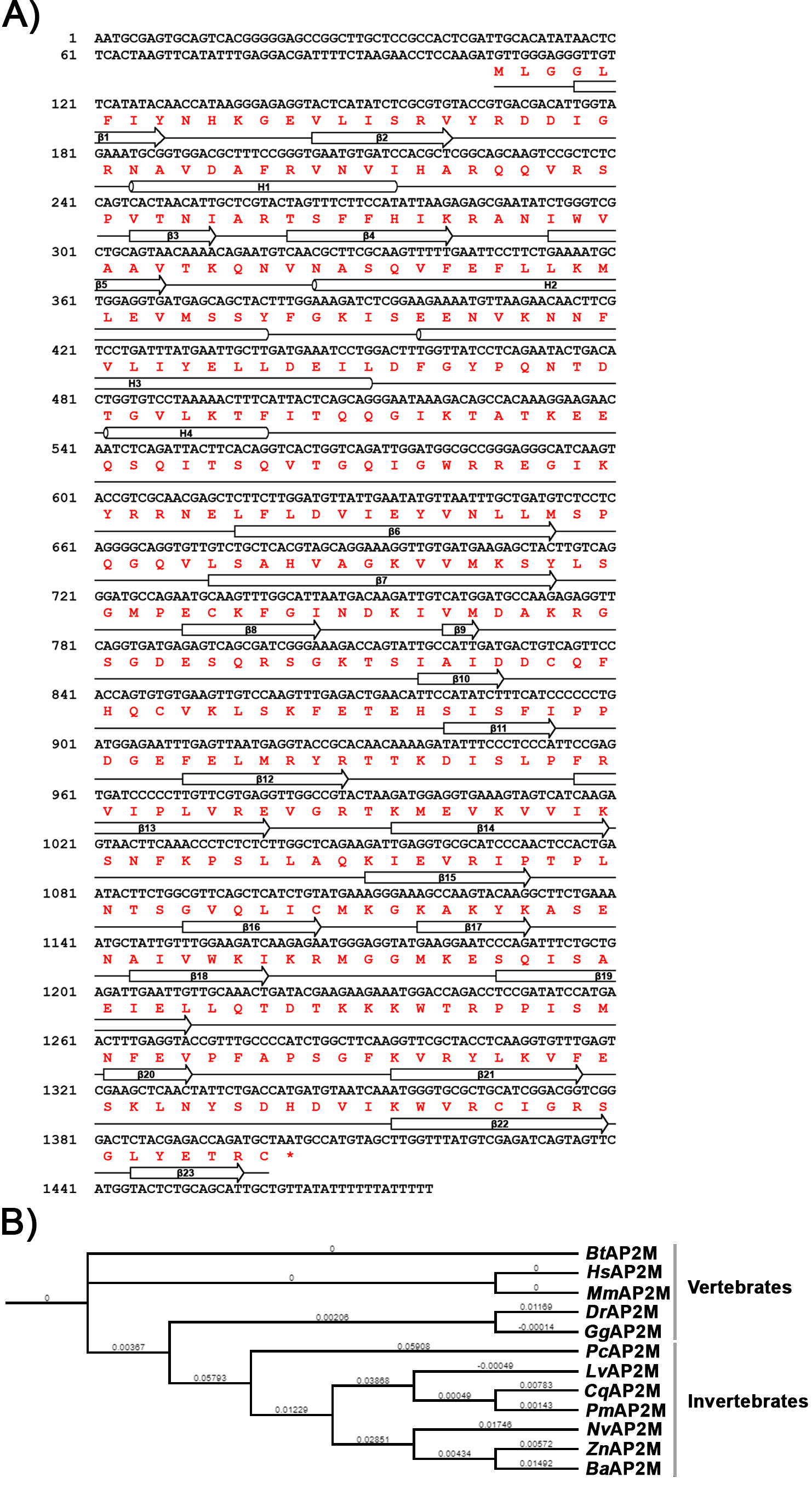


**Fig. S2** Analysis of nucleotide and amino acid sequences of *Pm*AP-2μ

(A) Nucleotide and amino acid sequence of *Pm*AP-2μ. Start codon is in a box. Secondary structure prediction was carried out using Jpred4 (53).

(B) Phylogenetic tree of AP-2μ from invertebrates and vertebrates. Sequences of AP-2μ were obtained from The National Center for Biotechnology Information (NCBI) and phylogenetic tree was generated by iTOL. AP-2μ are from *Bos taurus* (*Bt*APM, acc. NO.AAI02984.1); *Homo sapiens* (*Hs*AP2M, acc. NO.BAA09762.1); *Mus musculus* (*Mm*AP2M, acc. NO.AAH56352.1); *Danio rerio* (*Dr*AP2M, acc. NO.AAH49515.1); *Gallus gallus* (*Gg*AP2M, acc. NO.CAG30997.1); *Pomacea canaliculata* (*Pc*AP2M, acc. NO.XP_025081017.1); *Penaeus vannamei* (*Lv*AP2M, acc. NO.XP_027231007.1); *Cherax quadricarinatus* (*Cq*AP2M, acc. NO.ALP46597.1); *Penaeus monodon* (*Pm*AP2B); *Nicrophorus vespilloides* (*Nv*AP2M, acc. NO.XP_017771815.1); *Zootermopsis nevadensis* (*Zn*AP2M, acc. NO.XP_021913836.1); *Bicyclus anynana* (*Ba*AP2M, acc. NO.XP_023936830.1)


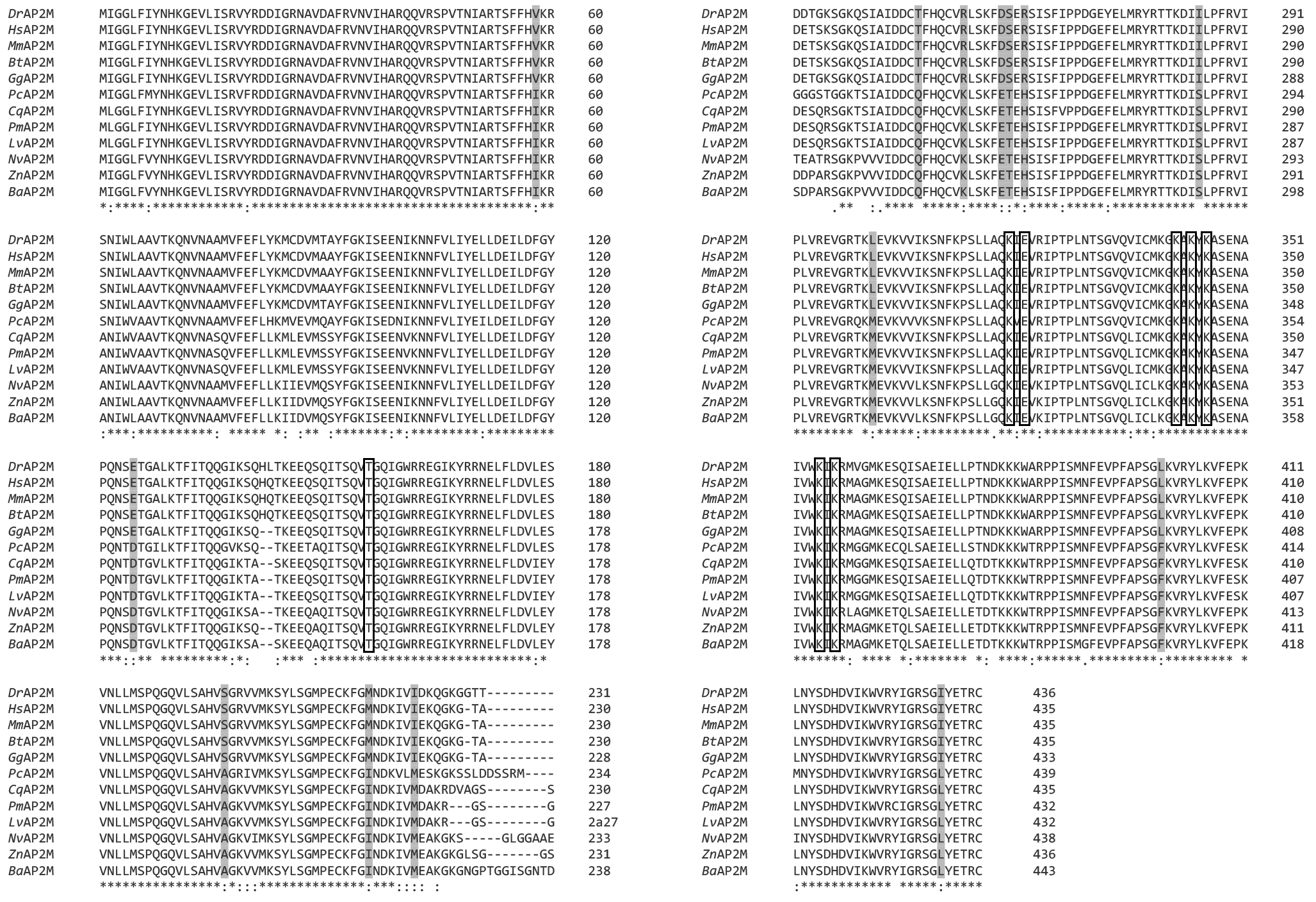


**Fig. S3** Multiple sequence alignment of AP-2μ from invertebrates and vertebrates. AP-2μ are from *Danio rerio* (*Dr*AP2M, AAH49515.1); *Homo sapiens* (*Hs*AP2M, BAA09762.1); *Mus musculus* (*Mm*AP2M, AAH56352.1); *Bos taurus* (*Bt*AP2M, AAI02984.1); *Gallus gallus* (*Gg*AP2M, CAG30997.1); *Pomacea canaliculate* (*Pc*AP2M, XP_025081017.1); *Cherax quadricarinatus* (*Cq*AP2M, ALP46597.1); *Penaeus monodon* (*Pm*AP2M); *Penaeus vannamei* (*Lv*AP2M, XP_027231007.1); *Nicrophorus vespilloides* (*Nv*AP2M, XP_017771815.1); *Zootermopsis nevadensis* (*Zn*AP2M, XP_021913836.1); *Bicyclus anynana* (*Ba*AP2M, XP_023936830.1). An asterisk indicates amino acid identity, and (.) and (:) indicate amino acid similarity.

**Table S1** Nucleotide sequences of the primers

| Primer name | Sequence (5’ – 3’) | Experiment |
| --- | --- | --- |
| 5'-RACE*Pm*AP-2m-R | CGGCGCCATCCAATCTGACCAGTGA3 | RACE |
| *Pm*AP-2mNcoI-F | CCATGGATGTTGGGAGGGTTGTTCA | cloning |
| *Pm*AP-2mXhoI-R | CTCGAGGCATCTGGTCTCGTAGAGTCC | cloning |
| *Pm*AP-2bEcoRI-F | GAATTCCATGACAGACTCAAAGTACTTCA | cloning |
| *Pm*AP-2bXhoI-R | CTCGAGCTTTCTCATTTTACATGCCATTT | cloning |
| knAP-2bT7-F | GGATCCTAATACGACTCACTATAGG CAACCAGCAGCAAGCACCACAG | double-stranded RNA synthesis |
| knAP-2bT7-R | GGATCCTAATACGACTCACTATAGG TGGTGTGCTTGAGGAGCCAATG | double-stranded RNA synthesis |
| knAP-2b-F | CAACCAGCAGCAAGCACCACAG | double-stranded RNA synthesis |
| knAP-2b-R | TGGTGTGCTTGAGGAGCCAATG | double-stranded RNA synthesis |
| knGFPT7-F | GGATCCTAATACGACTCACTATAGG ATGGTGAGCAAGGGCGAGGA | double-stranded RNA synthesis |
| knGFPT7-R | GGATCCTAATACGACTCACTATAGG TTACTTGTACAGCTCGTCCA | double-stranded RNA synthesis |
| knGFP-F | ATGGTGAGCAAGGGCGAGGA | double-stranded RNA synthesis |
| knGFP-R | TTACTTGTACAGCTCGTCCA | double-stranded RNA synthesis |
| EF1-α-F | GGTGCTGGACAAGCTGAAGGC | real-time RT-PCR |
| EF1-α-R | CGTTCCGGTGATCATGTTCTTGATG | real-time RT-PCR |
| *Pm*AP-2b-qRT-F | TCAAGAACAGCGTGGATGTG | real-time RT-PCR |
| *Pm*AP-2b-qRT-R | AATACCATCCGCATTCAGATTG | real-time RT-PCR |
| *Pm*STAT-qRT-F | TATATCCGAATGTGCCTAAG | real-time RT-PCR |
| *Pm*STAT-qRT-R | ATAGTTTGTGGTGTGTTGGG | real-time RT-PCR |
| *Pm*Spätzle-qRT-F | TAAGCAAGGAGCAGGAAGAG | real-time RT-PCR |
| *Pm*Spätzle-qRT-R | TGGCATACACCACATCTGAG | real-time RT-PCR |
| *Pm*Dorsal-qRT-F | TCACTGTTGACCCACCTTAC | real-time RT-PCR |
| *Pm*Dorsal-qRT-R | GGAAAGGGTCCACTCTAATC | real-time RT-PCR |
| *Pm*Relish-qRT-F | TCTCCAGGTGAGCACTCAGTTGGC | real-time RT-PCR |
| *Pm*Relish-qRT-R | GCTGTAGCTGTTGCTGTTGTTGAG | real-time RT-PCR |
| ALF*Pm*3-F | CCCACAGTGCCAGGCTCAA | real-time RT-PCR |
| ALF*Pm*3-R | TGCTGGCTTCTCCTCTGATG | real-time RT-PCR |
| *Pm*DOME-qRT-F | CTCAGGCTATGTTTCTCAGGATTCA | real-time RT-PCR |
| *Pm*DOME-qRT-R | CACGGCAGTTCCTTTATGGTCT | real-time RT-PCR |
| WSSV-IE1-F | GCTAGGGATGTGACTTTC | real-time RT-PCR |
| WSSV-IE1-R | TGCACCTACACGCATTAC | real-time RT-PCR |

The underline sequence indicates the recognition site of the restriction enzymes used for gene cloning. The double-underline indicates the T7 promotor sequence.

**
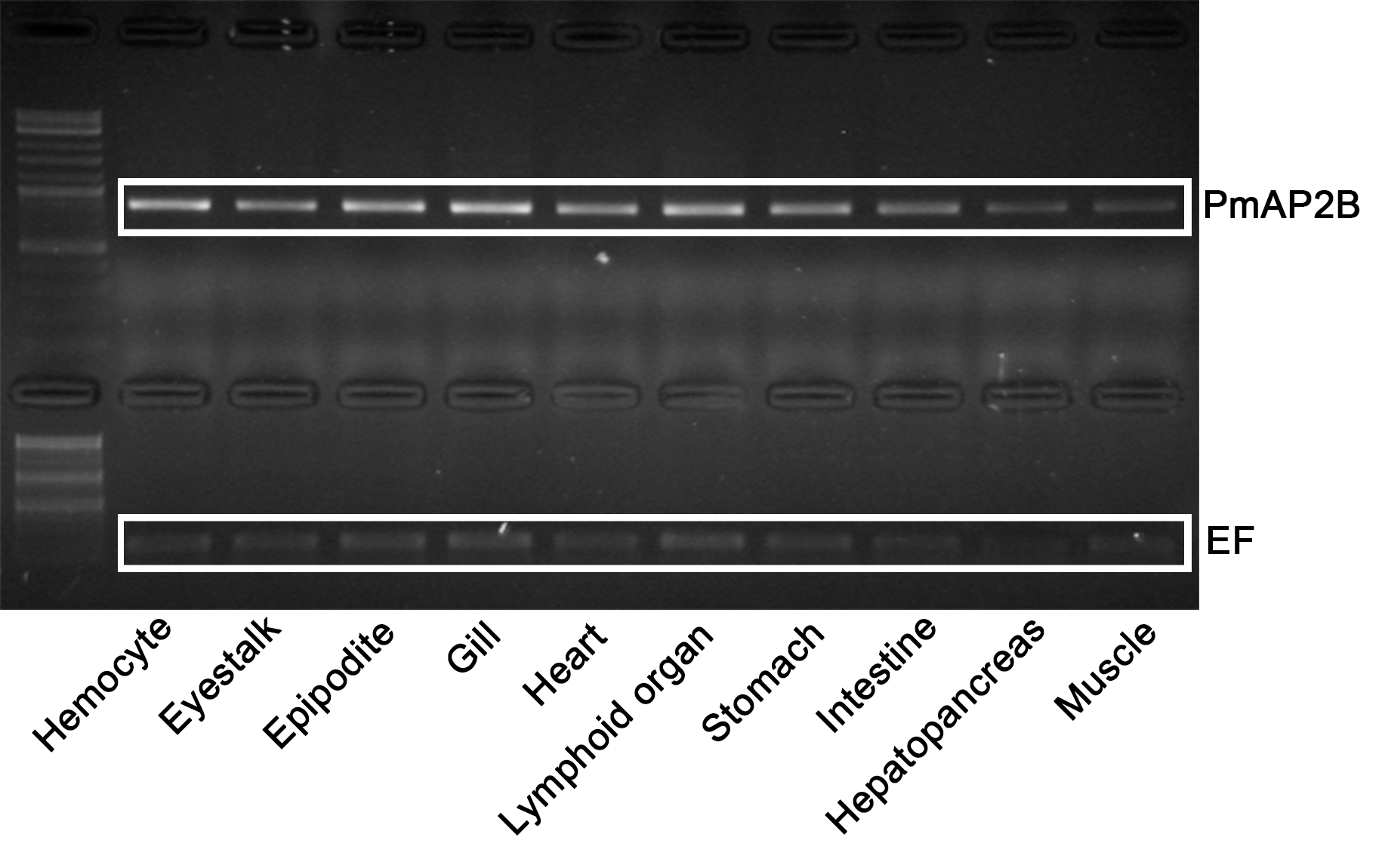
**

**Fig. 4A**

**
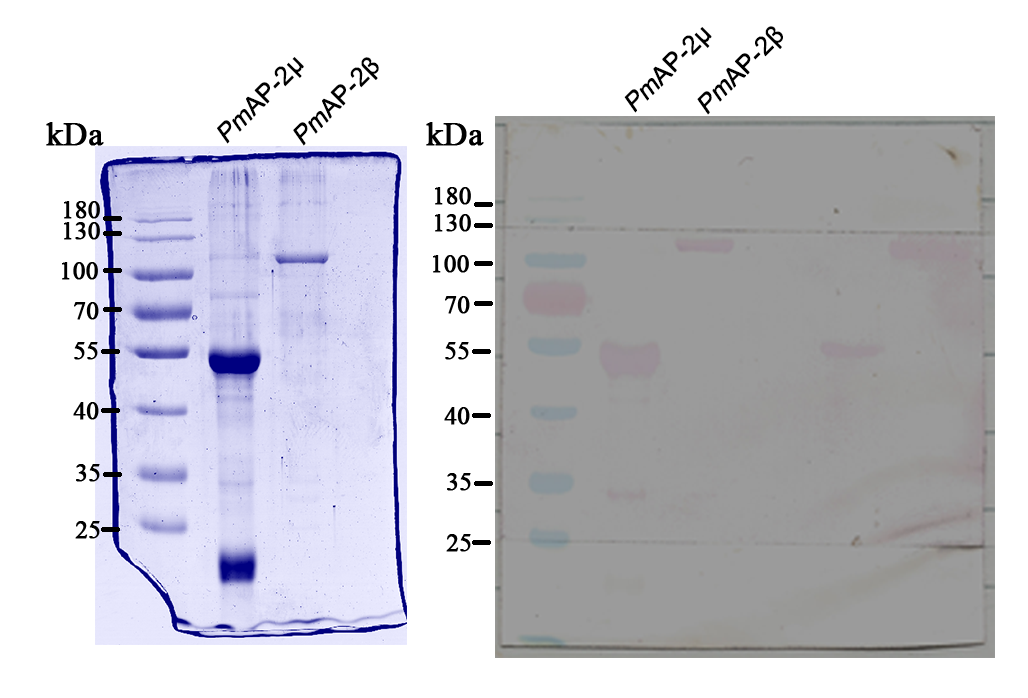
**

**Fig. S1**
